# Failure of psychiatric referrals from the pediatric emergency department

**DOI:** 10.1186/1471-227X-7-12

**Published:** 2007-08-15

**Authors:** Jacqueline Grupp-Phelan, Sergio V Delgado, Kelly J Kelleher

**Affiliations:** 1 Division of Emergency Medicine, Cincinnati Children's Hospital Medical Center, Cincinnati Ohio, USA; 2 Division of Child Psychiatry, Cincinnati Children's Hospital Medical Center, Cincinnati Ohio, USA; 3 Columbus Children's Research Institute, Columbus Children's Hospital, Columbus Ohio, USA

## Abstract

**Background:**

Recognition of mental illness in the pediatric emergency department (PED) followed by brief, problem oriented interventions may improve health-care seeking behavior and quality of life. The objective of this study was to compare the frequency of mental health follow up after an enhanced referral compared to a simple referral in children presenting to the PED with unrecognized mental health problems.

**Methods:**

A prospective randomized control trial comparing an enhanced referral vs. simple referral in 56 families of children who were screened for mental health symptoms was performed in a large tertiary care PED. Children presenting to the PED with stable medical problems were approached every fourth evening for enrollment. After consent/assent was obtained, children were screened for a mental health problem using both child and parent reports of the DISC Predictive Scales. Those meeting cutoffs for a mental health problem by either parent or child report were randomized to 1) simple referral (phone number for mental health evaluation by study psychiatrist) or 2) enhanced referral (short informational interview, appointment made for child, reminder 2 days before and day of interview for an evaluation by study psychiatrist). Data analysis included descriptive statistics and Chi-Square test to calculate the proportion of children with mental health problems who completed mental health follow-up with and without the enhanced referral.

**Results:**

A total of 69 families were enrolled. Overall 56 (81%) children screened positive for a mental health problem as reported by either the child (self report) or mother (maternal report of child mental health problem). Of these, 33 children were randomized into the enhanced referral arm and 23 into the simple referral arm. Overall, only 6 families with children screening positive for a mental health problem completed the psychiatric follow up evaluation, 2 in the enhanced referral arm and 4 in the simple referral arm (p = .13).

**Conclusion:**

Children screened in the ED for unrecognized mental health problems are very unlikely to follow-up for a mental health evaluation with or without an enhanced referral. Understanding the role of ED based mental health screening and the timing of an intervention is key in developing ED based mental health interventions.

## Background

Mental health problems in children presenting for medical care are increasing [1,2]. Despite poor health outcomes among affected children, recognition and treatment of mental health problems in children is inadequate [2-4] Moreover, once recognized by the health care system, children with mental health problems are often not effectively engaged into the mental health system or other treatment systems [5-7].

Mental health problems are highly prevalent in emergency department (ED) settings [8,9]. The pediatric emergency department represents a vulnerable and high-risk population. Because many such high risk youth do not have regular care, the emergency department represents a potential opportunity for the case identification and referral of mentally ill children[10,11]. Examples of successful similar ED-based behavioral brief interventions have been demonstrated in children presenting with alcohol and substance abuse, post-suicide management, smoking cessation and head injury follow-up [12-21]. All of these successful ED-based interventions include both an identification component along with expedited or coordinated referral to follow-up specialty services.

We hypothesize that identification of mental health problems in the ED followed by brief, problem oriented interventions and referral may improve on health-care seeking behavior and quality of life for the child. Central to our proposed intervention is an enhanced referral into mental health services. No study to date has compared an enhanced referral for specialty mental health services to a simple referral in children who screen positive for mental health problems in the emergency department setting. We were interested in testing whether an enhanced referral (i.e. easy access to a mental health evaluation and encouragement to go) would improve engagement into services for those youth with previously unrecognized mental health problems. Specifically, we were interested in whether, compared to simply giving a phone number for follow up, families in the enhanced intervention would complete the mental health follow-up plan more frequently. We also explored the barriers for not completing the follow up evaluation once a child screened positive for a mental health problem.

## Methods

We conducted a prospective randomized control comparison of an enhanced referral compared to a simple referral into mental health services for children who screened positive for a mental health problem. The trial was conducted in the emergency department of Cincinnati Children's Hospital Medical Center from June 2004 through November 2004 and was approved by the institutional review board.

### Subjects

**Figure 1:**
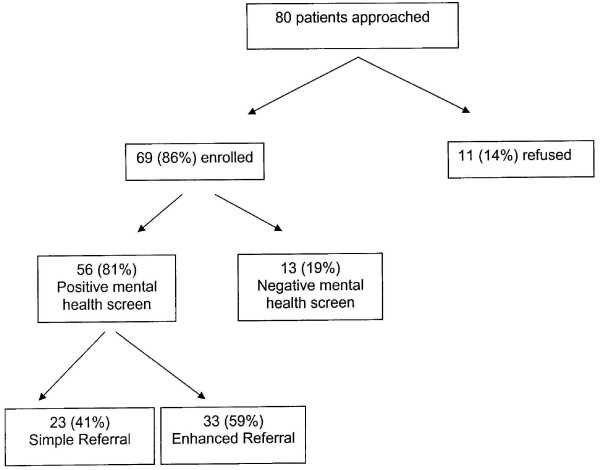
Sampling Schema.

Eighty children and their parents were approached for participation in the study of which 69 (86%) were enrolled. Consistent with our previous data in this high risk population [22], 56 children of the 80 (70%) screened positive for at least one mental health problem. Twenty three families were randomized into the simple referral group and 33 into the enhanced referral group (Figure 1). Children in both groups were similar with respect to child age, race, and insurance status. Children in the enhanced referral group were more likely to be male compared to the simple referral group.

All children between the ages of 4 and 18 who presented to the emergency department with urgent but stable medical problems were approached. Only residents of Hamilton County, the county encompassing the Children's Hospital, were approached to facilitate any potential long term mental health needs. After consent/assent was obtained, children were screened for mental health problems using the Diagnostic Interview Schedule for Children Predictive Scales [23]. Children who screened positive by either parent or child report were randomized into two groups: simple vs. enhanced referral for a comprehensive follow up mental health evaluation. The major outcome variable was completion of the follow-up mental health evaluation (treatment engagement).

Every fourth evening we approached the next available family based on triage number. Children were included if they were between the ages of 4–18 years and spoke English. Because <1% of our families did not speak English, sample size for non-English speaking families would have been so low as to provide unstable estimates of effect; thus the decision was made to exclude this population. Families without a telephone, cell phone or contact number were also excluded (this is less than 1% of our population- in a previous ED study 99% of families could provide a cell phone or contact number). Access to a phone was important due to the nature of the enhanced referral which required phone contact in the intervention group. During the consent procedure, families agreed to be contacted by a research assistant to be eligible for the study.

### Randomization

The patients were randomly assigned into the two groups using a random numbers table. Once a family consented and if their child screened positive for a mental health problem, the next number on the random numbers table was used to determine allocation into the intervention or comparison groups. Because we did not block randomize and the study was prematurely terminated, the two comparison groups were not equal in number based on the random numbers generated.

### Study intervention

The enhanced referral intervention involved a structured referral by a trained research assistant. The enhanced referral included a brief description of the mental health problem found and the importance of following through on the mental health follow-up. For example, if the child screened positive for depression, the research assistant would explain that "Your child screened positive for symptoms of depression, or feeling blue. Although this is a preliminary screen, it is important for your child to follow up with our mental health expert to see if this is really a problem for her. Not treating a child with depression can lead to problems at home, with friends and at school". A follow-up mental health appointment to the study psychiatrist (S.D.) was made for the family and a reminder phone call was made the day before and the day of the scheduled visit. The comparison group received the number to the same psychiatrist without a scheduled appointment or pre-visit phone reminder. Both groups were told that the comprehensive follow up evaluation would be timely and would not be charged to the family, thus taking access and payment barriers out of the reasons for non-engagement. All patients were given an evaluation within 1–2 weeks of the original visit (or for comparison families within 1–2 weeks of when they called the study psychiatrist for follow-up). Comparison families had the same ease of access into services compared to intervention families once they called the intake number.

### Measurement of disorders

Children ages 4–18 and their parents were given the Diagnostic Interview Schedule for Child Predictive Scale (DPS) [23] which uses the methodology of self-report questionnaires as a first stage assessment prior to second stage diagnostic evaluations. Using a set of questions derived from the lengthier NIMH-DISC [24], the DPS can accurately determine subjects who can safely be spared further diagnostic inquiry and can be used to identify cases of specific DSM-III-R disorders with excellent efficiency [23]. This 80-item instrument measures the presence of mental illness during the last 4 weeks (current point prevalence). Children self-reported from ages 9–18, and mothers reported for children between 4 and 9 years of age. Youth and parent informants often report differing key symptoms of a disorder. For this reason, both reports were solicited. A screen was considered positive if the child or parent report was positive.

### Outcome

The dependent variable of treatment engagement was defined as attendance to the follow up comprehensive mental health evaluation. Longer term outcomes were beyond the scope of this study.

### Barriers to engagement

Families randomized into the enhanced referral group who did not come for their comprehensive mental health evaluation were contacted by telephone after the study was halted to be interviewed about the barriers to follow-up. This portion of the study was approved by an amendment to the IRB after the finding of unexpectedly low treatment engagement. Barriers to treatment engagement were ascertained by a semi-structured telephone interview. Intervention families were asked a series of open ended questions regarding reasons for not making their appointments.

### Analyses

Rates of treatment engagement were examined by cross-tabulation, odds ratios and Chi Square based on intent to treat analysis. A power calculation was performed using a significance level of 0.05%, a 2 sided test, and a proportion of .4 following up in the simple referral group compared to a follow-up proportion of .6 in the enhanced referral group. We calculated that 94 children would need to be enrolled in each group to detect this difference with a power of .8 between treatment and comparison groups. Due to the failure to engage families in treatment, the study was discontinued after the 80^th ^family was approached (see below).

## Results

Families enrolled in the study were similar with respect to age. Families in the enhanced referral arm were more likely to be African American and male although this did not reach statistical significance. (Table 1) Families in the enhanced referral group were no more likely to engage in treatment (6%) compared to the simple referral families (18%) (Odds Ratio 0.29%: 95% CI 0.3–2.14). (Table 2)

**Table 1:** Demographic characteristics

	**Enhanced Referral (n = 33)**	**Simple Referral (n = 23)**	**P Value**
**Child Age **(mean yrs (Std Dev))	10.7 (4.3)	10.3 (4.2)	.73
**Race**			
Caucasian	14 (42%)	12 (52%)	
African American	17 (52%)	10 (44%)	
Other	2 (6%)	1 (4%)	.52
**Insurance Status**			
Medicaid	18 (54%)	10 (43%)	
Commercial	0	1 (4%)	
HMO	13 (40%)	8 (35%)	
Other (Self, none)	2 (6%)	4 (17%)	.45
**Sex **n (%)			
Male	22 (67%)	12 (52%)	
Female	11 (33%)	11(48%)	.41

**Table 2:** Mental health follow up by type of referral

	**Enhanced Referral (n = 33)**	**Simple Referral (n = 23)**
Completed Follow up Visit*	2	4

*p-.18

Overall, only 6 families (2 in the enhanced referral group and 4 in the simple referral group) followed up for the comprehensive mental health evaluation. Because of the unexpectedly low number of families who returned for a mental health evaluation, the study was discontinued after the 80^th ^family was approached prior to reaching the a priori sample size target. The multivariable analyses to look at type of mental health problem as a predictor of treatment engagement were also not performed for the same reason.

The children of the six families who came for a follow up interview were ages 4, 4, 6, 14, 16 and 16 years. The medical discharge diagnoses were abdominal pain, eye pain, headache, impetigo, seizure and strep throat correspondingly. Four were male and 2 female. All 6 had Medicaid, HMO payers or self pay- none had commercial insurance. Five of the six were African American and one was Caucasian. Of the two children in the Enhanced Referral group (ages 4 and 16 years, both male, one Caucasian and one African American) one screened positive for ADHD alone and the other for generalized anxiety, obsessive compulsive, social anxiety, and manic depressive disorders. Of the four in the simple referral group, the most common mental health symptoms were ADHD, oppositional defiant disorder, social anxiety disorder and obsessive compulsive disorder. Overall, all but one child screened positive for multiple mental health problems.

Families reported barriers to treatment engagement that fell into four general categories: 1) Problems were not "bad" enough to do anything about; 2) Worries about labeling the child with a mental health problem; 3) Logistical Issues related to coming back for the evaluation; and 4) Concerns about being asked these questions in the ED setting. (Table 3)

**Table 3:** Themes related to not following up

General Themes	Examples
Problems were not "Bad enough"	• "I believe my child has an issue that needs to be addressed and feel that a follow up visit would help with this problem, but it is not "bad" enough to warrant a visit now".
	• "I do think [my child] had a problem but don't think it is necessary [to have a mental health evaluation].
Labeling	• "I felt uncomfortable with the sense of him having ADHD. I didn't want for him to be labeled." This same mother believed her child had issues that needed to be dealt with. She also thought a follow up visit would help.
Logistical Issues	• A few parents had logistical problems making it to an appointment during the school day hours.
	• One mother was a home daycare provider and needed someone to take over for her to get time to come. She didn't really think there was a problem that needed to be addressed and thought these were "teenager things, she is doing teenager things, that they get angry".
Feelings about these questions in the ED	• One mother was anxious about being asked all the questions while at the ED. She did not feel she understood the nature of the interviews well. She worried that the investigator's follow up call meant something was wrong. (Interestingly, this mother ended up coming in for an evaluation with her child once her concerns were addressed.)

## Discussion

This study demonstrated that even when access and service payment issues are absent an enhanced referral into mental health services for children who screen positive for a mental health problem in an inner city emergency department is not enough to engage these families into services. Access and financial barriers to the initial mental health evaluation were intentionally taken out of the equation by the study's design. Both groups had equal access to the study psychiatrist once the follow-up number was called however only ten percent of families with a child who screened positive for a mental health problem followed up for an evaluation. This represents a serious gap in mental health service delivery for those families at highest risk for unmet mental health needs.

The lack of engagement effect of the intervention may have been related to the limited scope of the enhanced referral and the use of a research assistant instead of a seasoned provider. Although the potential engagement barrier of access was removed, the use of a research assistant to provide the intervention and advice for follow up may not have carried the same weight as a health care professional. Adding the element of motivational interviewing by a mental health provider may have increased the percentage of families who followed up for a mental health evaluation.

Recent evidence supports a collaborative approach to mental health treatment in adults [25] with an integration of services into the primary care setting resulting in a significant increased engagement into services compared to an enhanced referral [26]. In addition, severity of mental illness is related to service engagement, with suicidal adults being more likely to engage into services compared to less severely depressed adults [26]. Our study had a large spectrum of mental health diagnoses, with approximately 60% of positive screens reporting impairment in activities of daily living along with symptoms thus potentially less "severe" than other studies where screening was not generalized and where the subjects mental health issues were previously unrecognized. Indeed, one of the major themes that emerged from the families who did not complete a follow up interview was that the "problems were not bad enough" to seek help. Future interventions will need to assess how impairment and severity interact with treatment engagement.

Understanding the steps that high risk families go through prior to accessing mental health care may also hold the key for engagement interventions. Our enhanced referral sample contained more African-Americans than the simple referral group. Although lower rates of referral completion has been demonstrated in the literature for African Americans [27,28], in this study five out of six of the families who followed up for care were African American. Nonetheless, integrating a culturally relevant familial context into engagement interventions in this underserved population will be important for future studies.

A larger proportion of children in the enhanced referral sample were male, and four of out five of the children who followed up were male. Given the large proportion of externalizing symptoms in this group, it is likely that our follow up group represents a similar pattern to that which we see in the hospital's outpatient psychiatric clinic- males are seen more often due to externalizing behaviors. The under representation of females in our follow up group may reflect the decreased propensity to get help due to internalizing behavior such as depression.

## Conclusion

Our families reported being open to a mental health evaluation however many parents felt that the problems were not yet "bad enough to do anything about". It is possible that help was obtained by families through less formal networks of mental health services, such as school guidance counselors or from friends and from church as has been documented previously, particularly in a predominantly African American population. In addition, the presence or absence of social supports may be directly in the causal pathway of treatment engagement, with engagement being positively correlated to more structured social supports [29]. These and other factors related to a family's readiness to seek treatment will be important in the design of interventions to enhance successful treatment engagement.

## Competing interests

The author(s) declare that they have no competing interests.

## Authors' contributions

JGP conceived of the study, led its design and drafted the manuscript. SD carried out the follow up psychiatric evaluations and edited the manuscript. KK participated in the design of the study and edited the manuscript. All authors read and approved the final manuscript.

## Pre-publication history

The pre-publication history for this paper can be accessed here:


